# shinySISPA: A web tool for defining sample groups using gene sets from multiple-omics data

**DOI:** 10.12688/f1000research.13934.2

**Published:** 2018-06-15

**Authors:** Bhakti Dwivedi, Jeanne Kowalski

**Affiliations:** 1 Winship Cancer Institute , Emory University, Atlanta, GA, 30322, USA; 2 Department of Biostatistics and Bioinformatics, Emory University, Atlanta, GA, 30322, USA

**Keywords:** Sample profile, Gene set profile, Genomics, Integrated analysis

## Abstract

As opposed to genome-wide testing of several hundreds of thousands of genes on very few samples, gene panels target as few as tens of genes and enable the simultaneous testing of many samples.  For example, some cancer gene panels test for 50 genes that can affect tumor growth and potentially identify treatment options directed against the genetic mutation.  The increasing popularity of gene panel testing has spurred the technological development of panels that test for diverse data types such as expression and mutation.   Once samples are tested, there is the desire to examine clinical associations based on the panel and for this purpose, one would like to identify, among the samples tested, which show support for a molecular profile (e.g., presence of mutation with increased expression) versus those samples that do not among the genes tested.  With user-specified molecular profile of interest, and gene panel data matrices (e.g., gene expression, variants, etc.) that define the profile, shinySISPA (Sample Integrated Set Profile Analysis) is a web-based shiny tool to define two sample groups with and without profile support based on our previously published method from which clinical associations may be readily examined. The shinySISPA can be accessed from http://shinygispa.winship.emory.edu/shinySISPA/.

## Introduction

Unlike gene set profiling, sample profiling is a challenge due to the heterogeneity between, and within the tumor patient samples. Identification of homogenous groups of samples or molecular subtypes is commonly approached using clustering methods (e.g., Handl *et al.*, 2005; Kowalski *et al.*, 2016; Monti *et al.*, 2003; Șenbabaoğlu *et al.*, 2014; Verhaak *et al.*, 2010). Whether or not the sample groups are meaningful and the clustering stable, requires additional testing, is highly subjective, limited to examining changes in a single data type, and often require removal of genes or samples to obtain the desired results. While clustering tools such as TNBC subtyping ( Chen *et al.*, 2012; Lehmann *et al.*, 2011) are convenient for subtype discovery and sample classification, they are restricted to studying a specific cancer tissue and data type, within the context of established expression signature profiles. Although these methods may prove useful in certain case, there is a need for a basic tool that can identify sample groups using any combination of genomic data types based on a gene or gene set and molecular profile of interest. Some examples of gene sets may be derived from a specific biological process, network, gene enrichment analysis, a gene panel, etc. A molecular profile is a series of increasing or decreasing changes among diverse data types operating on a given gene set. For example, a gene mutation with expression is a molecular profile of increased variant support with increased levels of expression. The shinySISPA is a web tool developed to implement the novel method, SISPA ( Kowalski *et al.*, 2016), for defining samples with similar molecular profiles based on a user input gene set and data types. SISPA does not impose analytical distribution assumptions on the data, and is scalable to define samples that support a general profile defined by any combination of genomic data types applied to any number of genes.

## Methods

### Implementation

SISPA is written in the R programming language ( R project) and the shiny web application framework is implemented using the Shiny R package ( Chang *et al.*, 2016).

The tool is hosted on a 64bit CentOS 6 server ( http://shinygispa.winship.emory.edu/shinySISPA/) running the Shiny Server program designed to host R Shiny applications. This tool has been extensively tested on Windows 7 and Mac Pro 10 operating system with firefox and google chrome browser. Given a dataset of 377 samples and 16 genes under the two-feature analysis, it took three seconds to obtain shinySISPA defined sample groups and less than a second to generate the waterfall plot. The time it takes to generate sample profile diagnostic plots depends on the number of genes in a set; it took less than 10 seconds for 16 genes in both sets of a two-feature analysis. As a note, speed at which results are generated is also dependent on the internet connectivity.

### Operation

**Figure 1.**
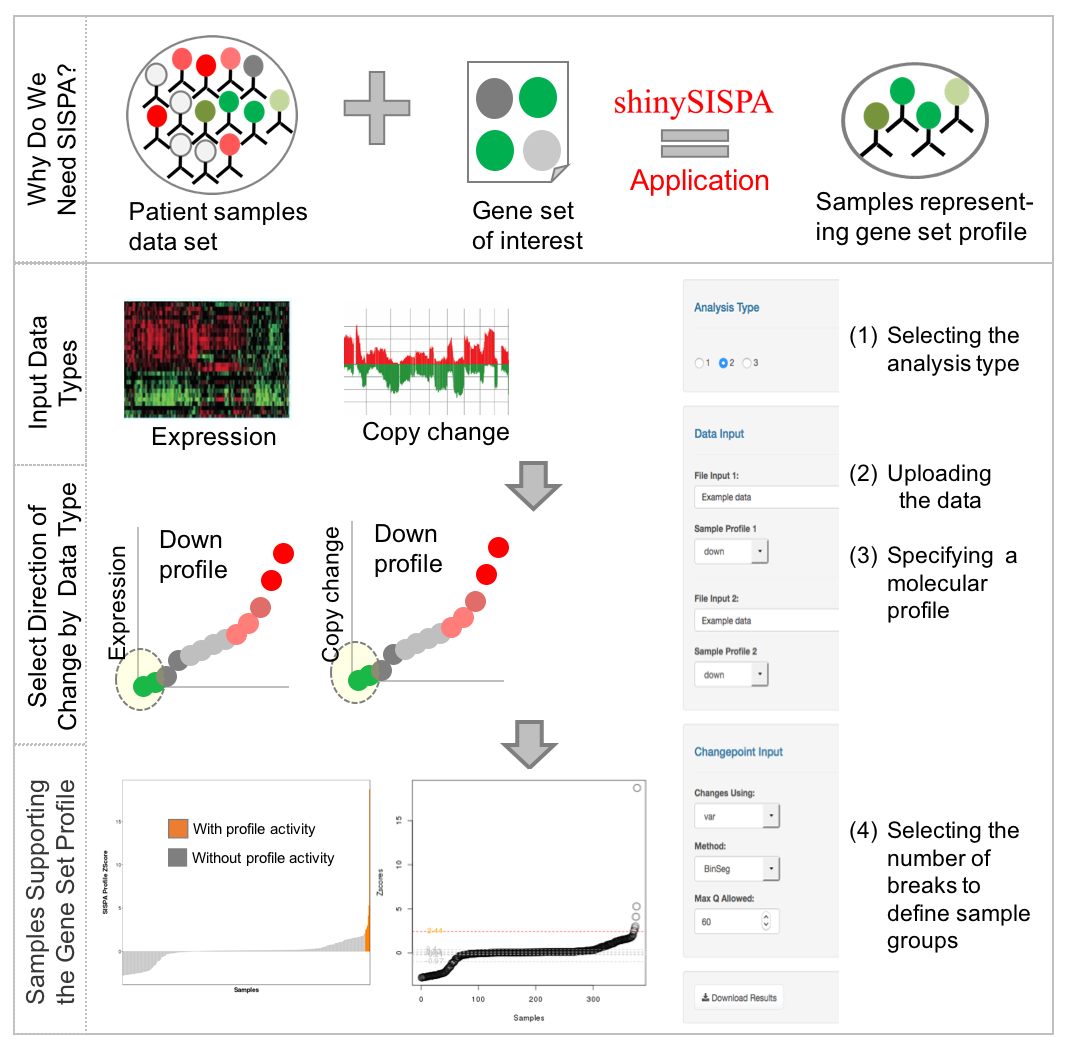
A schematic representation of shinySISPA workflow for a two-feature analysis. Here, we define samples supporting the molecular profile of decreased gene expression and copy loss. The tool requires user selection of analysis type, user upload of data types on samples and gene sets, and specification of a profile to output the samples supporting that profile. The samples are selected based on a change point model applied to composite (among features and genes), within-sample z-scores. A waterfall plot of profile activity is output with samples selected in orange as showing the most support for the profile.

The tool workflow consists of four basic inputs as shown in Figure 1:

(1) *Selecting the analysis type*. User selects a single-, two-, or three-feature analysis, where a feature corresponds to a specific data type (e.g., expression, methylation, mutation, copy number variation) and thus, a single-feature analysis refers to use of a single data type, while a two-feature uses a combination of two data types and so forth.

(2) *Uploading the data.* User inputs the data for each feature containing the genes and samples of interest. The same samples are required for each feature, though the gene sets may differ between features.

(3) *Specifying a molecular profile.* A molecular profile is a series of increasing (“up”) or decreasing (“down”) genomic changes within each feature. In Figure 1, a profile of decreased expression with decreased copy number is input.

(4) *Selecting the number of breaks to define sample groups*. User can specify the change point detection method ( Killick *et al.*, 2016) for finding optimal break points in the distribution of computed composite (among features) z-scores within samples ( Kowalski *et al.*, 2016; see Supplementary File 1).

The results are output in four separate tabs:

(1) *Input Data.* Summarizes the user input data in terms of the input number of genes, number of samples, and box plot distribution by data type.

(2) *SISPA Results.* Outputs the table of defined sample groups with their gene set enrichment score for the selected analysis type and molecular profile of interest. The scatter plot on the right displays all the change points detected within the data-set, samples falling in the topmost change point are the samples with the profile activity. The frequency plot at the rightmost bottom represents the distribution of the number of samples with and without the profile activity.

(3) *Waterfall Plot.* To visualize the sample groups that correlate with the profile of interest. Samples with the profile activity have the highest score and are shown in orange filled bars, while samples without the profile activity are shown with grey-filled bars.

(4) *Sample Profile.* Represents the diagnostic plots to visualize the distribution of the user-input data overall by the identified sample groups. It also allows the users to view data distribution for a selected gene in the set within each data type to assess what genes in particular satisfy the profile versus samples without profile.

All results generated during the process are directly downloadable on the user’s local computer. A detailed manual with tool settings are provided in the Supplementary File 1. Upon forming such sample groups, one may readily examine the effect of a profile on various clinical and biological clinical outcomes.

## Use case

We applied shinySISPA to profile newly diagnosed multiple myeloma (MM) patients for decreased gene expression and copy number based on a GISPA (Gene Integrated Set Profile Analysis)-derived gene set characterizing the IgH translocation in the MM cell lines ( Kowalski *et al.*, 2016). The t(14;16) translocation is known to be associated with poor prognosis in MM. By applying the shinySISPA tool to the t(14;16) characterized gene set profile, we were able to translate cell line profiles to patient profiles. Using the IA6 release of Multiple Myeloma Research Foundation (MMRF) CoMMpass study, we downloaded data from 377 newly diagnosed patients at pre-treatment with available clinical outcomes, RNA-Seq expression, and DNA-copy number variations from the MMRF Research Gateway portal. Based on our two-feature analysis, 7 of the 370 MM patients were defined with profile activity ( Figure 1) by identifying changes in variance using change point v2.2.2. Furthermore, we used CASAS ( Rupji *et al.*, 2017) to compare survival curves of the identified two sample groups for downstream clinical interpretation. We found seven samples with profile activity to be significantly ( *P*<0.0001) associated with poor survival as compared to the 370 samples without the profile activity (HR = 9.81; 95% CI = (3.39, 28.37)).

## Conclusion

We have demonstrated the utility of our shinySISPA tool in translating cell line characterized gene sets molecular profile to patient profiling ( Kowalski *et al.*, 2016); however, one can use any *a priori*-defined gene sets with any combination of molecular data for identifying samples with a similar gene set profile. The introduction of a change point model to select samples with profile support offers a more objective approach than with clustering methods. With only a gene set and a combination of data types from the same samples, the tool is widely applicable to many settings. For example, shinySISPA may be used to define patients based on known drug targets and pathways, or to identify patients that may be at risk for poor prognosis based on known prognostic markers.

## Data and software availability

The example sample data used to demonstrate shinySISPA workflow is available on the web-version and with the package source code at: https://github.com/BhaktiDwivedi/shinySISPA.

The shinySISPA software is available at http://shinygispa.winship.emory.edu/shinySISPA/

The stand-alone version of SISPA is available at https://www.bioconductor.org/packages/SISPA/.

Archived source code as at the time of publication: https://doi.org/10.5281/zenodo.1164284 ( Dwivedi & Kowalski, 2018)

License: shinySISPA is available under the GNU public license (GPL-3)

## Supplementary material

Supplementary File 1: The shinySISPA manual
