# Supplementary material for: shinySISPA: A web tool for defining sample groups using gene sets from multiple-omics data
Source: F1000Res. 2018 Jun 15;7:213. Originally published 2018 Feb 22. [Version 2] doi: 10.12688/f1000research.13934.2 (PMC6097349; doi:10.12688/f1000research.13934.2)
Supplement: Supplementary file 1 [file f1000research-7-16536-s0000.tgz › 29da12c2-1cf9-44b5-8bcf-419f1e57c90d.docx]

**The shinySISPA Manual**

**Introduction**

shinySISPA is a web-based tool intended for the researchers who are interested in defining samples with similar gene set enrichment profiles. The shinySISPA tool is based on a novel SISPA method published in our previous paper (Kowalski, *et al*., 2016). SISPA defines samples as with and without profile *activity*, on average, among genes defined as a set, by applying a change point model (Killick, et. al., 2016) to a composite, between features zscore obtained by adding or subtracting individual sample zscores between features, depending on their corresponding profiles (Fig 1; see Kowalski, *et al*., 2016 for method details). The individual sample zscores are computed using the Bioconductor R package GSVA (Hänzelmann, *et al*., 2013) for more than or equal to three genes.

*
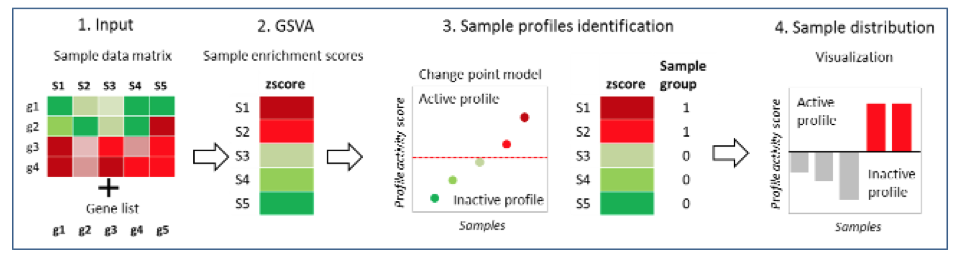
*

**Figure 1**. Schematic representation of the SISPA method

**Running shinySISPA on a local computer:**

1. Download and install R or RStudio (version 3.3.2. or later) from <https://cran.r-project.organd>
2. Open R and install the below required packages:

> install.packages(c("shiny", "GSVA", “genefilter”, "changepoint", "data.table", “ggplot2”,

"plyr"))

1. Users can run shinySISPA locally using the source code available from the GitHub: <https://github.com/BhaktiDwivedi/shinySISPA>, by typing the below commands in R console:

> library(shiny)

> runApp("shinySISPA")

1. Users can also download and run the app from GitHub directly using:

> shiny::runGitHub('shinySISPA', 'BhaktiDwivedi')

**Example Sample Dataset**

We have used publically available multiple myeloma (MM) patient data from the ongoing Multiple Myeloma Research Foundation (MMRF) CoMMpass clinical trial (<https://www.themmrf.org/)> to identify sample groups using GISPA derived gene set characterizing IgH translocation, t(14;16) in the MM cell lines (Kowalski, *et.al.,* 2016). Data from 377 patients with available clinical outcome, RNA-seq expression, and copy number change at pre-treatment was downloaded from the MMRF researcher gateway portal (<https://research.themmrf.org)> based on the IA6 release of this trial. The expression is normalized log2 transformed count data, while copy number is the mean segment value. The data is available to download from GitHub (<https://github.com/BhaktiDwivedi/shinyGISPA>). Users can also access and analyze this dataset by choosing the “Example data” from the “Data Input” option under the two-feature Analysis Type on the web-interface.

**Getting Started**

1. **Select the analysis type**

Click on options under “Analysis Type” to select a single feature or two-feature analysis. Here feature is defined by a data type (e.g., expression, somatic mutations).

An example of single-feature analysis could be identifying samples with increased (or decreased) expression profile within the defined gene set signature, while a two-feature analysis could be based on a combination of any two data types, e.g., identifying samples that exhibit decreased gene expression and decreased copy change.

1. **Upload the Input Data**

Upload the input data file given the selected data type from (1). User uploads the input data file for genes of interest and profile to define samples on within the data type. The gene sets can be defined based on prior knowledge derived from either biological processes, pathways, biomarkers discovery, genomic analysis, or integrated gene set analysis (e.g., GISPA). A *profile* is a genomic change of either increase (“up”) or decrease (“down”) within a specific feature or data type.

File format requirements:

- Maximum file size limit of up to 200 MB.
- ASCII formatted tab-delimited file, where each row represents a gene and each column a sample.
- Here is an example of a user uploaded ‘File Input’ for one data type analysis. First column is gene id followed by samples data as shown in the screenshot below:

- Here is an example of user uploaded ‘File Input 1’ and File Input 2’ for two data type analysis. For each input data file, first column is gene id followed by samples data as shown in the screenshot below:

**

- When running shinySISPA on two data types, the samples must overlap between the two input data files. The two input data files must have the same exact number of samples and sample (or column) names. Rows that corrospond to genes (probes, variants, or any other id) may or may not be the same.
- A minimum of at least one gene and ten samples are required.
- No duplicated column names or duplicated row names are allowed and analysis will be stopped.
- Genes (or rows) with zero variance across all samples will be excluded from the analysis.

Snapshot of shinySISPA using Single-feature analysis:

Snapshot of SISPA using Two-feature analysis:

1. **Select the Changepoint Method**

User can specify/modify the change point detection method (Killick R, *et al*., 2016), i.e., to find the optimal break points within the estimated profile sample score (Kowalski, *et al*., 2016). The changes can be found using mean(“mean”), variance (“var”) or both (“meanvar”) with the user-specified changepoint method (“AMOC”, “BinSeg”, “PELT”, or “SeqNeigh”) given the allotted maximum number of change points (“Max Q Allowed”). Note that the number of change points identified may differ for the same dataset depending on the change point R package version installed on the system. Currently we are running changepoint version 2.2.2 on our hosting server.

1. **Result**

The results are output in four tabs:

- 1. Input Data
  2. SISPA Results
  3. Waterfall Plot
  4. Sample Profile
  5. **Input Data**

Summarizes the user input data in terms of the input number of genes (or rows), number of samples (or columns), and number of genes (or rows) with zero variance among all the samples to be excluded from the analysis. Data for only the first five genes overall samples is shown and visualized as boxplot.

- 1. **SISPA Results**

Table of defined sample profiles with their gene set enrichment score for the selected data type and sample profile of interest. The enrichment scores for each sample are computed using the zscore method (Hänzelmann, et. al., 2013). The score statistics is rank ordered by the user selected profile (e.g., up or down) for each sample. A change point model (Killick, et. al., 2016) is then applied to the sample scores to identify groups of samples that show similar gene set profile.

Samples assigned a value of 1 in the *‘sample_group’* column are the samples with the user selected profile activity, while samples assigned a value of “0” are the samples without the profile activity. The results table can be searched, sorted, and filtered by any of the four columns. The scatter plot on the right displays all the change points detected within the data-set, samples falling in the topmost change point are the samples with the profile activity. The frequency plot at the rightmost bottom represents the distribution of the number of samples with and without the profile activity.

- 1. **Waterfall Plot**

Visual representation of all samples sorted by their zscores. Samples with the profile activity have the highest score and are shown in orange filled bars, while samples without the profile activity are shown with grey-filled bars. For any selected sample profile, whether “up” or “down”, the orange-filled bars with the highest scores are the most desirable and represent samples with profile activity.

- 1. **Sample Profile**

Represents the distribution of the user-input data overall by the sample groups with and without the profile activity. This enables to assess whether or not, on an average, the entire gene set defines the sample groups by the user selected sample profile. Within the “Sample Profile” tab, user also have the option to select a “Gene to Display” from the “Choose Options” drop down menu on the rightmost panel, to see the input data distribution by each sample sorted within each sample profile. This enables the user to identify the gene(s) that are most distinguishing among samples with and without the profile activity.

1. **Save Results**

User can download the results as one excel file by clicking on the “Save” button on the left panel. The download includes table and pdf plots of the results.

**Citation**

Please cite the SISPA method as: Kowalski J, Dwivedi B, Newman S, Switchenko JM, Pauly R, Gutman DA, *et al*. Gene integrated set profile analysis: a context-based approach for inferring biological endpoints. Nucleic Acids Research. 2016;44(7):e69.

**References**

Hänzelmann, S., Castelo, R. and Guinney, A. GSVA: gene set variation analysis for microarray and RNA-seq data. *BMC Bioinformatics* 2013;14(7).

Killick R, Eckley IA. changepoint: An R Package for Changepoint Analysis. J Stat Softw. 2014;58(3):1-19.

Kowalski J, Dwivedi B, Newman S, Switchenko JM, Pauly R, Gutman DA, *et al*. Gene integrated set profile analysis: a context-based approach for inferring biological endpoints. Nucleic Acids Research. 2016;44(7):e69.
