# Supplementary material for: shinySISPA: A web tool for defining sample groups using gene sets from multiple-omics data
Source: F1000Res. 2018 Jun 15;7:213. Originally published 2018 Feb 22. [Version 2] doi: 10.12688/f1000research.13934.2 (PMC6097349; doi:10.12688/f1000research.13934.2)
Supplement: Peer review history

# Peer review: Referee response for version 2

**DOI:** 10.5256/f1000research.16536.r36536

**Published:** 2018-08-15

**Authors:** Younghee Lee

**Affiliations:** 1 Departments of Biomedical Informatics, University of Utah School of Medicine, Salt Lake City, UT, USA

This work proposes a web-based tool allowing to identify a subgroup of samples that share similar molecular profiles based on gene expression information and visualize the results in various plots. It is written in R program language. This tool would be useful for easily dealing with clinical data and genomic data.

 One weakness is to only define two sample groups but not multiple groups.

I have read this submission. I believe that I have an appropriate level of expertise to confirm that it is of an acceptable scientific standard.

# Peer review: Referee response for version 2

**DOI:** 10.5256/f1000research.16536.r35118

**Published:** 2018-06-15

**Authors:** Yun Zhang

**Affiliations:** 1 Department of Biostatistics and Computational Biology, University of Rochester, Rochester, NY, USA

The authors clarified and answered my previous concerns, and I have no further comments.

I have read this submission. I believe that I have an appropriate level of expertise to confirm that it is of an acceptable scientific standard.

# Peer review: Referee response for version 1

**DOI:** 10.5256/f1000research.15147.r32660

**Published:** 2018-04-16

**Authors:** Yun Zhang

**Affiliations:** 1 Department of Biostatistics and Computational Biology, University of Rochester, Rochester, NY, USA

The authors introduce a Shiny application that are developed as a user-friendly tool for conducting omics-data analysis with their published methodology work. The article provides detailed description of the inputs and outputs for the shiny application, and shows working example for the use of this application. This article is index-able with some necessary modifications.

 The following are my major comments.

1. Before introducing the shiny application, the authors should provide a brief summary of their methodology work, including clear definitions of important terminologies and a description of their model. It would greatly help the readers to understand the contents followed subsequently.
  1. With a glimpse on the methodology paper, I found the terms such as “molecular profile”, “feature” are defined misleadingly in this article.
  2. What is a change point model?
2. For the input element (4), where to select the number of breaks? Is it the max Q allowed? What is the max Q? What are the options for “Changes Using” in the bottom right of Figure 1?
3. For the output element (2), it is better to include a figure with the description.
4. In the “Use case” section,
  1. Why GISPA is used? What’s the relation of GISPA and SISPA?
  2. How many genes are used? Is it 16?
  3. Where are the 7 patients in Figure 1? Are they the orange bars?
  4. What is “using change point v2.2.2”?
  5. “We found seven samples with profile activity to be significantly ( *P*<0.0001) associated with poor survival as compared to the 300 samples without the profile activity (HR = 9.81; 95% CI = (3.39, 28.37)).” Previously mentioned, there are 377 patients. Why are 70 samples missing?
5. For the reproducibility of the work, please upload a default dataset in the shiny application, so that the readers/users can replicated the analysis for the first time.

Also, I provide my minor comments below.

1. Please consider a thorough revision of the English writing for this scientific article. There are a number of grammatical errors, and sentences do not flow smoothly. Please avoid using colloquial words in scientific writing.
2. Please modify the legend of Figure 1. In the brackets “(shown in grey)”, it is hard to locate where it is referring to since there are many grey colors in the figure. Also, please add labels to the subfigures that are referred in the text.

I have read this submission. I believe that I have an appropriate level of expertise to confirm that it is of an acceptable scientific standard, however I have significant reservations, as outlined above.

# Response

**Published:** 2018-06-01

**Authors:** Bhakti Dwivedi

**Affiliations:** Emory University, USA

Referee Report 16 Apr 2018

Yun Zhang, Department of Biostatistics and Computational Biology, University of Rochester, Rochester, NY, USA 

**Approved with Reservations**

The authors introduce a Shiny application that are developed as a user-friendly tool for conducting omics-data analysis with their published methodology work. **The article provides detailed description of the inputs and outputs for the shiny application**, and shows working example for the use of this application. This article is index-able with some necessary modifications.

The following are my major comments.

1) Before introducing the shiny application, the authors should provide a brief summary of their methodology work, including clear definitions of important terminologies and a description of their model. It would greatly help the readers to understand the contents followed subsequently.

**Response: **We have provided a reference to our methods paper that includes detailed information on the SISPA approach.  In this paper, our focus is upon introducing the application of the method in terms of tool development and implementation by providing detailed examples and information on data input and output/results.  Considering this focus, along with space constraints, we have opted to not repeat the already published method description and instead, reference it. 

1. With a glimpse on the methodology paper, I found the terms such as “molecular profile”, “feature” are defined misleadingly in this article.

**Response: **We have intentionally used the terms “molecular profile” and “feature” in the same context as in the published methods papers, whereby “molecular profile” refers to change of either increase (“up”) or decrease (“down”) and “feature” refers to a specific data type (e.g., expression, methylation, copy number change).  We have also provided examples of the term “molecular profile” to further clarify the context.

In this paper:

      “A “feature” corresponds to a specific data type (e.g., expression, methylation, mutation, copy number variation) and thus, a single-feature analysis refers to use of a single data type, while a two-feature uses a combination of two data types and so forth.” (pg# 3 last paragraph, under *Selecting the analysis type*)

      “A “molecular profile” is a series of increasing (“up”) or decreasing (“down”) genomic changes within each feature…” (pg# 4 second paragraph, under *Specifying a molecular profile)*

In the published methodology paper:

    “The Cancer Genome Atlas (TCGA) nomenclature ( https://tcga-data.nci.nih.gov/tcga/dataAccessMatrix.htm) that references a specific data type (RNA-Seq expression, DNA CpG methylation, etc.) as a feature.”

    “A profile is defined by specifying *a priori*, a change of either increase or decrease within each of the features..”

        2.  What is a change point model?

**Response: **A changepoint model is a method for identifying changepoints within data. It is a published method. Please see below references to the changepoint method and the changepoint R package for details:

- Killick R and Eckley IA (2014). “changepoint: An R Package for Changepoint Analysis.” *Journal of Statistical Software*, **58**(3), pp. 1–19. http://www.jstatsoft.org/v58/i03/.
- Killick R, Haynes K and Eckley IA (2016). *changepoint: An R package for changepoint analysis*. R package version 2.2.2, https://CRAN.R-project.org/package=changepoint.

2) For the input element (4), where to select the number of breaks? Is it the max Q allowed? What is the max Q? What are the options for “Changes Using” in the bottom right of Figure 1?

**Response:** Yes, the allotted maximum number of breaks is specified using the “Max Q Allowed”.

“In input element (4), users can modify the Changepoint Input (Killick R, *et al*., 2016) to find the optimal break points within the estimated profile sample score (Kowalski, *et al*., 2016). The changes can be found using mean(“mean”), variance (“var”) or both (“meanvar”) with the user-specified changepoint method (“AMOC”, “BinSeg”, “PELT”, or “SeqNeigh”) given the allotted maximum number of change points (“Max Q allowed”). Note that the number of change points identified may differ for the same dataset depending on the change point R package version installed on the system. Currently we are running changepoint version 2.2.2 on our hosting server...”

Please see pg# 6 of the supplementary file 1 for the details.

3) For the output element (2), it is better to include a figure with the description.

**Response: **The output elements (“Input Data” “SISPA results”, “Waterfall Plot” and “Sample Profile”) screenshot including the figures are explained in detail in the supplementary file 1. Please see pg# 7-11 under “Result” Section.

4) In the “Use case” section,

1. Why GISPA is used? What’s the relation of GISPA and SISPA?

**Response:**

   GISPA (Gene Integrated Set Profile Analysis) is a method designed to define gene sets with similar, a priori specified molecular profile. While, SISPA (Sample Integrated Set Profile Analysis) is a method designed to define sample groups with similar gene set a priori specified molecular profile. Both GISPA and SISPA method are published in Nucleic Acid Research (Kowalski et al., 2016; PMID: 26826710).

   GISPA was used to identify genes with decreased expression and decreased copy change molecular profile in a multiple myeloma cell line with IgH translocation. This gene set is published in the methodology paper Nucleic Acid Research (Kowalski et al., 2016; PMID: 26826710).

   Here, we extracted RNA-seq expression, and copy number change data for GISPA derived gene set characterizing the IgH translocation on 377 newly diagnosed patients enrolled in the coMMpass clinical trial to define samples with a similar gene set profile, i.e., decreased expression with copy loss. This example data is provided with this paper.  Pg# 4 last paragraph under “Use case” describes the use of application of SISPA using GISPA derived gene sets.

       2. How many genes are used? Is it 16?

**Response:** Yes. The number of genes in the expression and copy number variation data is 16.

       3. Where are the 7 patients in Figure 1? Are they the orange bars?

**Response: **The patients with and without profile activity are highlighted in “Samples Supporting the Gene Set Profile” labeled section of the Figure 1. Yes, the 7 patients are highlighted in orange-filled bars.

       4. What is “using change point v2.2.2”?

**Response: **“using change point v2.2.2”? means that we have used changepoint R package version 2.2.2.

       5. “We found seven samples with profile activity to be significantly ( *P*<0.0001) associated with poor survival as compared to the 300 samples without the profile activity (HR = 9.81; 95% CI = (3.39, 28.37)).” Previously mentioned, there are 377 patients. Why are 70 samples missing?

**Response:** We have corrected the typo, please see page# 4 and 5, last paragraph. It is 7 of 370 patients.

“Based on our two-feature analysis, 7 of the 370 MM patients were defined with profile activity ( Figure 1) by identifying changes in variance using change point v2.2.2. Furthermore, we used CASAS ( Rupji *et al.*, 2017) to compare survival curves of the identified two sample groups for downstream clinical interpretation. We found seven samples with profile activity to be significantly ( *P*<0.0001) associated with poor survival as compared to the 370 samples without the profile activity (HR = 9.81; 95% CI = (3.39, 28.37)).”

5) For the reproducibility of the work, please upload a default dataset in the shiny application, so that the readers/users can replicated the analysis for the first time.

**Response:** All users can access and analyze the example dataset (i.e., default dataset) used in the paper by choosing the “Example data” from the Upload Input option on the web-interface.  The data is also available to download from GitHub ( https://github.com/BhaktiDwivedi/shinySISPA). Users are able to obtain the same exact results, i.e., samples with and without profile activity using the current default settings implemented in shinySISPA web tool.

Also, I provide my minor comments below.

1.  Please consider a thorough revision of the English writing for this scientific article. There are a number of grammatical errors, and sentences do not flow smoothly. Please avoid using colloquial words in scientific writing.

**Response: **We have reviewed the manuscript and do not identify any such problem. If the reviewer still feels strongly about it, we kindly request specific examples to be cited from the text.

2. Please modify the legend of Figure 1. In the brackets “(shown in grey)”, it is hard to locate where it is referring to since there are many grey colors in the figure. Also, please add labels to the subfigures that are referred  in the text.

**Response: **We have addressed and incorporated these changes. Please see updated Figure 1 and Figure legend.

 

- Is the rationale for developing the new software tool clearly explained?

Yes

- Is the description of the software tool technically sound?

Partly

- Are sufficient details of the code, methods and analysis (if applicable) provided to allow replication of the software development and its use by others?

No

- Is sufficient information provided to allow interpretation of the expected output datasets and any results generated using the tool?

Partly

- Are the conclusions about the tool and its performance adequately supported by the findings presented in the article?

Partly

**Competing Interests:** No competing interests were disclosed.

**I have read this submission. I believe that I have an appropriate level of expertise to confirm that it is of an acceptable scientific standard, however I have significant reservations, as outlined above.**
